# Interpretation and approximation tools for big, dense Markov chain transition matrices in population genetics

**DOI:** 10.1186/s13015-015-0061-5

**Published:** 2015-12-30

**Authors:** Katja Reichel, Valentin Bahier, Cédric Midoux, Nicolas Parisey, Jean-Pierre Masson, Solenn Stoeckel

**Affiliations:** INRA, UMR1349 Institute for Genetics, Environment and Plant Protection, 35650 Le Rheu, France

**Keywords:** Discrete stochastic model, Sparse approximation, Eigenvector, Network analysis, Population genetics, Compositional data, de Finetti diagram

## Abstract

**Background:**

Markov chains are a common framework for individual-based state and time discrete models in evolution. Though they played an important role in the development of basic population genetic theory, the analysis of more complex evolutionary scenarios typically involves approximation with other types of models. As the number of states increases, the big, dense transition matrices involved become increasingly unwieldy. However, advances in computational technology continue to reduce the challenges of “big data”, thus giving new potential to state-rich Markov chains in theoretical population genetics.

**Results:**

Using a population genetic model based on genotype frequencies as an example, we propose a set of methods to assist in the computation and interpretation of big, dense Markov chain transition matrices. With the help of network analysis, we demonstrate how they can be transformed into clear and easily interpretable graphs, providing a new perspective even on the classic case of a randomly mating, finite population with mutation. Moreover, we describe an algorithm to save computer memory by substituting the original matrix with a sparse approximate while preserving its mathematically important properties, including a closely corresponding dominant (normalized) eigenvector. A global sensitivity analysis of the approximation results in our example shows that size reduction of more than 90 % is possible without significantly affecting the basic model results. Sample implementations of our methods are collected in the Python module *mamoth*.

**Conclusion:**

Our methods help to make stochastic population genetic models involving big, dense transition matrices computationally feasible. Our visualization techniques provide new ways to explore such models and concisely present the results. Thus, our methods will contribute to establish state-rich Markov chains as a valuable supplement to the diversity of population genetic models currently employed, providing interesting new details about evolution e.g. under non-standard reproductive systems such as partial clonality.

**Electronic supplementary material:**

The online version of this article (doi:10.1186/s13015-015-0061-5) contains supplementary material, which is available to authorized users.

## Background

Natural systems often possess inherently discrete states in space, time or both. Atoms, molecules and cells, organs, individuals, populations and taxa usually appear as distinct entities; along the time axis, the radiation cycles we use as the basis for atomic clocks, neuronal action potentials, developmental stages in an organisms life cycle, generations and the revolutions of the earth around the sun are examples for similar patterns.

Modeling these discrete systems as such can have advantages over continuous approximations. One of the earliest examples comes from thermodynamics [1], where heat emission spectra could only be predicted correctly if energy “comes in packets”, known as “quanta”. This discovery led to the new field of quantum mechanics, which provided the necessary theory for understanding the photovoltaic effect [2], thus proving essential for the invention of solar cells. In biology, the re-discovery of Mendel's rules and thus of the “quantal” nature of genetic heritability, at about the same time as Planck’s famous speech, has had a similar impact on the study of evolution as the latter’s research has had on thermodynamics [3]. While most of the objects of biological research have long been recognised as discrete (e.g., the word *individual* literally means *not dividable*, a notion very similar to that of a *quantum*), we still struggle with understanding the processes, such as evolution, linking them to potential emergent properties (analogous to the physicists’ heat spectra) at higher levels. Mathematical models preserving the discrete nature of the biological system are thus an interesting field of study.

Markov chains are a classical framework for modeling state and time discrete stochastic systems. Based on the assumption that the modeled system is *memoryless* (Markov property, [4]), the basic model equation consists in multiplying a “start” vector, providing the state of the system at a given time, with a typically square “step” matrix. This matrix holds the transition probabilities, which depend on the model parameters and typically remain constant through time, between all possible states of the system within one time step. By analyzing the transition matrix, both the “short term” transient behavior and the “long term” limiting behavior of the model can be studied, thus putting the matrix at the center of attention for the biological interpretation of the results. Markov chains and other related forms of matrix-based models, such as Leslie models in population dynamics, are already widely in use (e.g. [5– 7]), yet in many cases the number of modeled states is comparatively small and/or a major part of the transitions are considered impossible. The latter property leads to many zeros in the transition matrix, which then becomes *sparse*, as opposed to a *dense* matrix where zeros are rare. Computationally, sparse matrices are advantageous since memory may be saved by storing only those values which are different from zero. Special algorithms exist to carry out standard operations (e.g. matrix multiplication) directly on matrices stored in a sparse format (e.g. [8, 9]).

In population genetics, state and time discrete Markov chains are known primarily by the example of the classic biallelic Wright-Fisher model [3], which uses a one-dimensional random walk to describe the evolution of allele frequencies under genetic drift. For a population of *N* diploid organisms, the states of the Markov chain correspond to each of the $2N+1$ possible combinations of counts of the two alleles that sum to the constant total 2*N*. Accordingly, a square transition matrix (assuming constant population size) would have $(2N+1)^{2}$ entries. As the number of states further increases both with the population size and the complexity of the underlying genetic system (number of alleles and loci, Table 1), the dynamics of allele frequencies in bigger populations are typically approximated by a continuous diffusion process based on the Fokker-Planck/Kolmogorov equations [5], or even by deterministic equations assuming an “infinite” population size (e.g. as for the derivation of the Hardy-Weinberg equilibrium, [10, 11]). An alternative approach is coalescence theory, which uses re-defined discrete states and a reversed continuous time scale to specifically approximate certain aspects of the original state and time discrete Markov chain (e.g. [12, 13]). While each of these approximations has its strengths and weaknesses (e.g. as discussed in [14, 15]), population genetic models that stay with the classic state and time discrete, chronological framework appear to be rare. One example is the model presented in [16]: an extension of a classic biallelic Wright-Fisher model, it is based on genotype rather than allele frequencies. This design appears better adapted for the study of partially clonal populations, but also results in a bigger state space (e.g. for two alleles, combinations of the counts of each of three genotypes rather than those of the two alleles). The technical effort of storing and manipulating the big, dense transition matrices essential to such a model hardly seems to merit the results, which in turn have to be extracted from a great amount of data; adapted methods for interpretation and storage size reduction appear to be missing.

**Table 1:** Examples of matrix size based on the Stoeckel-Masson model. Memory use is approximate and assumes 64-bit accuracy

*N*	$\mathcal {P}$	$\mathcal {L}$	$\mathcal {A}$	*g*	|*S*|	Memory use
20	2	1	2	3	231	420 KB
100	2	1	2	3	5151	205 MB
500	2	1	2	3	125,751	120 GB
1000	2	1	2	3	501,501	2 TB
20	4	1	2	5	10,626	865 MB
20	2	2	2	9	3,108,105	75 TB
20	2	1	4	10	10,015,005	730 TB
20	2	2	4	100	$9.8 \times 10^{20}$	$6.5 \times 10^{21}$ YB

In this article, we provide a set of methods for visualizing and interpreting both the transient and limiting behavior of population genetic models involving state-rich, irreducible, aperiodic and time-homogeneous Markov chains, based on the transition matrix and its dominant eigenvector, as well as a method for approximating a dense transition matrix by a sparse substitute. For the first part, we combine *de Finetti* diagrams [17] with network analysis, extending both concepts to provide clear and informative diagrams for the analysis of population genetic processes. For the second part, we use a predefined threshold (minimal percentage of information contained in the transition matrix) to keep only the more probable transient behavior of the model, while at the same time ensuring that mathematically important matrix properties are kept. The model presented in [16] serves as an example to illustrate our methods.

## Model example

The population genetic model of Stoeckel and Masson [16] describes the evolution of genotype frequencies based on a single locus with two alleles *a* and *A* in a fixed-size population of diploid, partially asexual organisms. States are defined as assignations of the *N* individuals in the population to the three possible genotypes (*aa*, *aA*, *AA*). The transition probabilities between the states depend on a symmetric mutation rate $\mu$ and a constant rate of asexual reproduction *c*, defined as the probability that an individual in the next generation was derived clonally from a single parent.

**Fig. 1:**
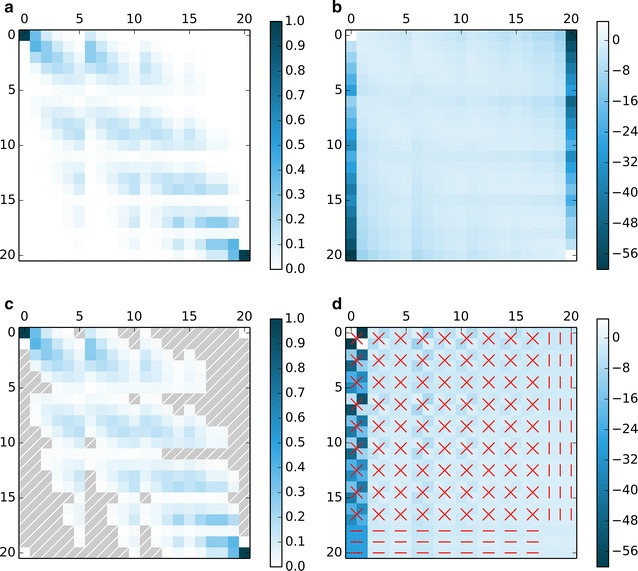
Heat maps of transition matrices for $N=5, \mu =10^{-6}, c=0.0$. **a** original probabilities, dense matrix **b**. logit(10) transformed probabilities, dense matrix **c**. Sparse approximate matrix of **a**, implicitly stored zero values in *hatched grey* **d**. As in **b**, with alternative state order, *red lines* connect identical values

**Fig. 2:**
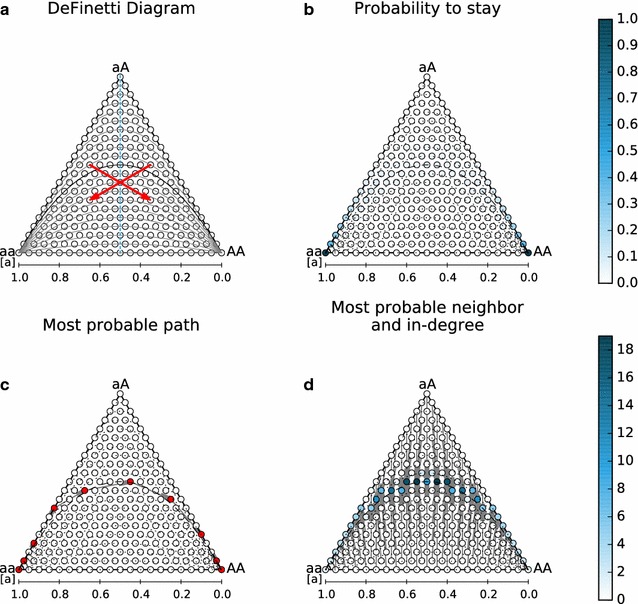
Network display of transition matrices for $N=20, \mu =10^{-6}, c=0.0$. **a**. *De Finetti* diagram showing symmetry *(dashed blue axis*, *red arrows* corresponding to identical probabilities) and $F_{IS}$ isocurves (*gray* and *black*). **b** $p_{stay}$ (node color), probability to stay at each node for one time step. **c** Most probable path connecting (N,0,0) to (0,0,N). **d** Most probable neighbors (directed edges) and in-degree (node color), i.e. for each node the most likely outbound transition at the next time step and the number of inbound most likely transitions from other states. Enlarged version in Additional file 2

Transition matrices *M* resulting from this model are generally square, due to the fixed population size (a common feature of many population genetic models, compare [3]). They also have a density of one—transitions between all states are possible in one step, although some of them (e.g. all individuals *aa* to all individuals *AA*) are very unlikely. The corresponding Markov chain is thus irreducible (single communicating class, no absorbing states) and aperiodic (period of all states equals one, same state possible in consecutive time steps). Since the mutation rate $\mu$ is symmetric, i.e. changes from *a* to *A* are just as likely as the inverse, *M* is also partially symmetric: if the transition probabilities from one particular state to all others have been calculated, swapping the names of all alleles also gives a correct result (compare Figs. 1, 2). The notation in this article assumes left-stochastic matrices (columns represent the transition probabilities from one state to all others and thus sum to one), which implies that the limiting behavior of the Markov chain is described by its transition matrices’ (normalized) right eigenvector *v* to the eigenvalue with the largest absolute value (and multiplicity one, [18]): one.

The number of states in this model, and thus the size of the transition matrix *M*, depends on the one hand on the population size and on the other hand on the complexity of the genomic system being modeled, in particular the number of different genotypes possible. For a given number of genotypes *g*, the cardinality of the state space *S* (respective number of rows and columns in the transition matrix) in a genotype-based discrete stochastic model is:

1 $$\begin{aligned} \left| {S}\right| = \left( \!\! {g \atopwithdelims ()N}\!\!\right) = \frac{\left( N + g-1 \right) !}{N! \cdot \left( g-1\right) !} \end{aligned}$$

From this equation it follows that the number of states increases exponentially with $1+ (g-1)/(N+1)$ for increasing *N* and with $1+ N/g$ for increasing *g*. For the number of possible genotypes, the ploidy level of the organism $\mathcal {P}$, the number of (partially linked) loci $\mathcal {L}$ and their respective numbers of alleles $\mathcal {A}_{i}$, with $i \in 1 \ldots \mathcal {L}$, need to be taken into account:

2 $$\begin{aligned} g = \prod _{i=1}^{\mathcal {L}} \left( \!\!{\mathcal {A}_{i} \atopwithdelims ()\mathcal {P}}\!\!\right) = \prod _{i=1}^{\mathcal {L}} \frac{\left( \mathcal {A}_{i} + \mathcal {P} -1 \right) !}{\mathcal {P}! \cdot \left( \mathcal {A}_{i}-1\right) !} \end{aligned}$$

Examples for the size of the resulting transition matrices are given in Table 1. From these numbers, it is clear that a realistic “base-by-base” model of a full genome is still far beyond the capacity of current computer technology; however, many cases (biallelic SNPs, unlinked loci or blocks of completely linked loci) can already be interpreted based on the very simple *one-locus/two-alleles* model [19]. It remains the dependence of |*S*| on the population size *N*, which is fortunately not so strong (for $N>g-1$).

To illustrate our methods, we will mostly use transition matrices derived for completely sexual populations ($c=0.0$), a case for which both transient and limiting behavior are generally known and interpretations can be easily verified [3, 17]. For the mutation rate, $\mu = 10^{-6}$ was chosen as a plausible value based on experimental estimates [20–23]. *N* is either 5 ($|S| = 21$), 20 ($|S| = 231$) or 100 ($|S| = 5 151$), for good visibility and easy reproducibility of the results. Our test of the sparse approximation method is based on the limiting distribution of $F_{IS}$, a population genetic parameter of wide interest (e.g. as discussed in [23] under the name *f*, or in [24]) that was also analyzed in the original article describing our model example [16]. For our example, the definition of $F_{IS}$ based on the allele $(\nu _{a}, \nu _{A})$ and genotype frequencies $(\nu _{aa},\nu _{aA},\nu _{AA})$ is:

3 $$\begin{aligned} F_{IS} = 1-\frac{\nu _{aA}}{2\,\nu _{a}\nu _{A}} = 1-\frac{\nu _{aA}}{2\,(\nu _{aa}+0.5\,\nu _{aA})(\nu _{AA}+0.5\,\nu _{aA})}. \end{aligned}$$

## Results

Working with big, dense transition matrices poses two connected problems: on the one hand, the storage size of the matrix may considerably slow down calculations or be altogether too big for the computer, on the other hand, the relevant information about the model may be difficult to extract from the great amount of data contained in the matrix. Visualization techniques for the interpretation of matrix data can, however, also help to find matrix properties which allow reducing the storage size, such as partial symmetry or the occurrence of many near-zero transition probabilities. We therefore start by describing the visualization techniques in the first part, and then move on to storage size reduction by sparse approximation in the second part of the results.

### Visualization

An intuitive first step in analyzing the transient behavior of a Markov chain model is a diagnostic visualization of the transition matrix. By summarizing results in an accessible way, the resulting diagram may ideally also provide a basis for direct biological interpretation. With one exception (landscape plot), all the following visualization methods are available using the functions *histogrid*, *histo3d* and *networkplot* (with its support function *percolation*) in the *mamoth* module; an example for the runtime of each method is given in Additional file 1.

#### Heat map

A heat map or histogram of the transition matrix, where the transition probabilities *p* are symbolized by color/ shade or height, is perhaps the easiest way to visualize it (Fig. 1). The resolution may be enhanced by an appropriate transformation of the range of values for *p*, for example by using a negative logarithm ($[0;1] \rightarrow [0; \infty ]$) or a *logit* transformation ($[0;1] \rightarrow [-\infty ; \infty ]$).

For big matrices, heat maps can be costly to produce (memory size) and are often still not very clear, due to the large number of cases. However, they may help to recognize basic patterns (symmetries, groups of similar/more strongly connected states etc.) of potential value for finding more adapted visualizations/numerical methods.

- In our example, the heat map shows that many of the transition probabilities in the matrix are, though not equal, very close to zero. After re-ordering the states, the partial symmetry of the matrix also becomes visible.

#### Network display

The duality between matrices and graphs (e.g. [7, 25]) provides an alternative for the visualization and mathematical analysis of either structure. In a graph $\mathcal {G(V, E)}$, the states of a Markov chain are thus represented as nodes/vertices $\mathcal {V}$ and the transitions as (weighted and directed) edges $\mathcal {E}$ connecting them, which is especially useful for sparse transition matrices.

For big, dense matrices, the number of edges in the resulting complete multidigraph (of edge multiplicity two) equals the number of entries in the transition matrix and is thus too big for easy interpretation. Concepts from network theory can be used to selectively display edges and summarize information about each state of the model system on the nodes. This leads to a variety of very clear synthetic representations constructed with different parameters and taking into account different time scales: from one generation (based on *M*) across *t* generations (based on $M^{t}$) up to the long-time equilibrium (dominant eigenvector of *M*, *v*).

To facilitate biological interpretation, we arranged the nodes of the network according to biological “meta data”. For our model example where states represent distributions of individuals on three genotypes (*aa*, *aA*, *AA*) under a constant population size (compositional data), we placed the nodes in a *de Finetti* diagram ([17], see Fig. 2), a specialized ternary plot for population genetics.

The following visualization techniques are based on selectively displaying the network’s edges:

*Most probable neighbor* This is the analog to a *nearest neighbor* if distances (edge weights) represent probabilities. For each state *i*, there are one or several states *j* which have the *highest* probability to be the destination of a transition in the next time step; tracing these connections gives the expectation for the one-step transient behavior of the model.

- In our example, the most likely state for the next generation (Fig. 2) is always on or very near to the Hardy-Weinberg Equilibrium, which is represented by the continuous black curve going through (1/4; 1/2; 1/4) in the diagram in Fig. 2a.

*Most probable path* This is the counterpart of a *shortest path* if distances (edge weights) represent probabilities. For each non-commutative pair of states *i* and *j*, there exists at least one series of consecutive edges connecting *i* to *j* along which the *product* of the edge weights is *maximal*. It can be determined by using an “ordinary” shortest path algorithm (e.g. [26, 27]) on a negative *log* transform of the transition matrix. The most probable path is the most likely trajectory of the model system to get from one state to another.

- In our example (Fig. 2), a change from a population with only the *aa* genotype to one with only the *AA* genotype would closely follow the Hardy-Weinberg curve.

*Flow threshold* Using the smallest probability along the most likely path between two nodes *i* and *j* as a threshold, very rare transitions can be excluded.

- In our example (Additional file 3), horizontal transitions along the base of the triangle, where no heterozygotes are produced despite of two homozygous genotypes being present in the population, would be excluded.

The following visualization techniques are based on changing the appearance of the network’s nodes:

*Degree* For each node in a graph representing a dense matrix, the number of incoming (*in-degree*) and outgoing (*out-degree*) edges is normally (approximately) equal to the number of nodes (matrix rows/columns). This method should therefore be used in connection with selective edge plotting and interpreted according to context.

- In our example (Fig. 2), the nodes with the highest in-degree are nearest neighbors to the largest number of nodes; if all states were equally likely at the current generation, those next to (0.25; 0.5; 0.25) on the Hardy-Weinberg curve would be the most likely in the next generation.

*Betweenness-centrality* Based on the same concept as the *most probable path*, this can be redefined as the number of *most probable paths* passing through each node when connections between each pair of nodes are considered. It can be derived in a similar way as the *most probable path*, by applying a standard algorithm developed for additive distances to a negative *log* transform of the multiplicative probabilities in *M*. Nodes with a high betweenness-centrality represent frequent transient states.

- In our example, these are all the states along the Hardy-Weinberg curve except for the fixation states (Additional file 4).

*Probabilities* For each state *i* in the Markov chain model, several probabilities can be calculated—and displayed on the nodes—to describe both the transient and limiting behavior:

$$p_{\text {stay}}$$
*probability to stay for one time step*
  $p_{\text {stay}}(i) = p_{i,i}$, the probabilities on the matrix diagonal; for each state *i* this is the probability that the system remains at state *i* for the next time step (“stickiness”). This probability allows the easy detection of (near-)absorptive states.
  In population genetics, the fixation states $$\lbrace (N;0;0),$$$$(0;0;N)\rbrace$$ are typical examples (Fig. 2).
$$p_{\text {out}}$$
*probability to leave in one time step*
  $p_{\text {out}}(i) = 1-p_{i,i},$ the column sums of the matrix without the diagonal; for each state *i* this is the probability that the system changes state at the next time step (“conductivity”). Being the opposite of $p_{stay}$, this probability allows the detection of states which are rarely occupied for consecutive time steps. In our example, these are the states where the population consists of an approximately even mixture of both homozygotes (central basis of the triangle) or only of heterozygotes (top of the triangle; Additional file 3). In contrast, the row sums of a left-stochastic matrix may exceed one and are thus not probabilities. As a result of the Markov property, a *probability to arrive* always depends on the state at the previous time step, which results in a number of possible definitions.
*p*(*i*|*j*) : *probability to arrive from state j in one time step*
  $p(i|j) = p_{j, i}, j \in S$, all probabilities in one column of the transition matrix; the probability distribution (mean, variance, skew according to arrangement of nodes) for transitions starting from one particular state. This allows the prediction of the most likely states for the next time step. In our example, the variance around the fixation states is much more limited than at the interior states of the triangle (Additional file 4).
$$p_{\text {in}}$$
*probability to arrive in one time step*
  $p_{\text {in}}(i) = 1/(|S|-1) \cdot \sum _j p_{j, i}$ for $i \ne j$, the row sums of the matrix divided by the number of other states; probability to arrive at state *i* if all previous states are equally likely. This shows states which are generally very likely destinations for one-step transitions. In our example, these are the states around the Hardy-Weinberg curve (additional file 3).
$$p_{\text {in}}^{\infty }$$
*probability to arrive in an infinite run*
  $p_{\text {in}}^{\infty }(i) = \sum _j p_{j, i} \cdot v_{j}$ for $i \ne j$, the sum over the element-wise product of eigenvector and matrix row, without the diagonal; probabilities to arrive at state *i* if the likelihood of the previous states is distributed according to the limiting distribution. This shows the states which are the most frequent destination of transitions in an infinite run of the model. In our example, these are the two states next to the fixation states where there is exactly one “foreign” allele (Additional file 4).
$$p^{\infty }$$
*limiting distribution/eigenvector-centrality*
  $p^{\infty }(i) = v_{i}$, the eigenvector; probability to find the system at state *i* after infinitely many time steps, or proportion of time spent in each state averaged over infinitely many time steps (limiting distribution). This is the prediction for the most likely states independently of the start state. As is well known for our example, these are the fixation states (Additional file 3).

*Expected time to first passage* To calculate the expected time to arrive at a certain (group of) states from any other, the “target” states are considered absorptive (first passage time, [7]). Based on the sub-matrix $M'$ including only the transition probabilities between non-target states, the times $t_{\text {target}}$ are

$$\begin{aligned} t_{\text {target}} = \mathbf {1}(I-M')^{-1} \end{aligned}$$

where $\mathbf {1}$ is a row vector of ones matching the dimension of $M'$ and *I* is the corresponding unit matrix. The first passage times of the target states are zero.

- For our example, plotting the expected time to the fixation states shows that it depends predominantly on the current state’s allele frequencies (Additional file 4).

#### Landscape plot

Combining length and direction of the transitions in the most probable neighbor plot (Fig. 2) gives a three dimensional “landscape” illustrating the most probable dynamics of the Markov chain, similar to the “gravity well” plots known from physics. The expected changes in the genotype frequencies are thus represented in a more intuitive fashion, by imagining the population as a small ball rolling on a “landscape” from “hills” to “valleys”. Elevations *h* are derived from the equality of potential and kinetic energy, which resolves to

$$\begin{aligned} h=d^2 \cdot 0.05 \end{aligned}$$

for a single time step, approximating gravitational acceleration by 10. For each model state/node, the distances *d* are given by the changes in genotype frequencies when moving to the most probable neighbor

$$\begin{aligned} d = \sqrt{(\Delta aa)^{2}+(\Delta aA)^{2}+(\Delta AA)^{2}}. \end{aligned}$$

The “landscape” is subsequently drawn as a triangular grid, using the elevation at each state/node as support. To improve readability, *h* can be rescaled by a constant factor and the landscape colored according to the relative elevation (taking the center of each triangle as reference). The resulting “landscape” shows only the (deterministic) expected dynamics of the Markov chain one could imagine the accompanying stochastic effects as an “earthquake”.

- In our example, the expected dynamics of the genotype frequencies show convergence to the Hardy-Weinberg equilibrium (Additional file 5).

*Note:* because of its dependence on a function or matrix specifying the distances between states, and on the triangular grid-like structure of the state space, this method is not included in the *mamoth* source code.

### Approximation

One major drawback of state-rich Markov chain models is that the transition matrix in its full form takes up a lot of memory (Table 1). Beside switching to one of the alternative model types mentioned in the introduction (diffusion approximation, coalescence process), there are multiple computational approaches to addressing this issue while keeping the original state and time discrete framework, including:

- *External memory:* the whole matrix is stored on a (sufficiently large) hard drive, only parts are loaded into active storage when needed (analogous to [28])
- *Iterative/selective matrix creation:* the whole matrix is never stored, only parts are created when needed (e.g. in combination with algorithms such as [29])
- *Lumping states based on model properties:* if a group of states has the same (sum of) transition probabilities leading into it and out of it to any other (group of) states and the same analytical meaning (e.g. same value of $F_{IS}$) they can be combined into one ([30, 31]); other algorithms of state aggregation, such as [32], lead to an approximation of the original matrix
- *Sparse approximation:* turning a dense matrix into a sparse matrix by approximating very small matrix elements to zero (e.g. as in [33, 34])

Which of the first two options is more appropriate depends both on the available hardware and the nature of the task: if the whole matrix is needed repeatedly, storing it will save the time to recalculate despite increased memory access times, but if calculating the matrix elements is fast, the matrix is needed only once or only some parts of the matrix (e.g. the *most probable neighbor* of each state) are needed, storing the matrix as a whole would be an unnecessary effort.

Because of the symmetry between the two allele frequencies in our model example, almost half of all states could be pairwise lumped, thus reducing matrix size to a little over a quarter of the original. The exception are the states on the symmetry axis of the *de Finetti* diagram (compare Figs. 1, 2), which do not have a “lumping partner”. Symmetry with respect to the allele frequencies is often found in population genetics models [3]. However, because of this dependency on model structure a size reduction algorithm based on lumping would not be applicable to non-symmetric extensions of the original model, e.g. with an asymmetric mutation rate or directional selection. Allele frequencies would have to be analyzed jointly, as the new states retain only the ratio of both; once lumped, “unpacking” the states becomes difficult.

The high number of very small values in the Markov chain transition matrix (Fig. 1) of our model example suggests that sparse approximation would be very effective. Moreover, as each column of the matrix corresponds to a probability distribution (constant sum of one) which becomes less uniform as the number of states/population size increases (the expected convergence to a multinormal distribution with variance proportional to 1/*N* is the underlying principle of the well-known diffusion approximation), the proportion of very small transition probabilities is likely to augment as the matrix size increases. While sparse approximation is independent of model-specific properties such as symmetry and does not change the states as such, it has the disadvantage of changing the actual content of the transition matrix, potentially leading to the loss of relevant properties such as left-stochasticity or irreducibility.

The sparse approximation algorithm we propose ensures that the resulting sparse matrix still has all the properties relevant to its function in the Markov chain model. Additionally, it can be executed iteratively so that the complete dense matrix need not be stored. The algorithm iterates over all columns of the transition matrix *M* and excludes (almost) all values which, in total, contribute less than a threshold value $s \in [0,1]$ to the column sum:

for all columns $C^i = M_{1\ldots |S|,i}$ with $i \in [1, |S|]$:

1. Create a permutation *R* of the row indices so that the corresponding entries are ranked according to size: $R \leftarrow \text {ordinalrank}(j\, |\, 1 \ge C^i_{j} \ge 0)$
2. Find the minimal rank (index of *R*) so the corresponding entries sum at least to the threshold value *s*: $r \leftarrow \text {min}(k)$ for $\sum _{R_1}^{R_k} C^i_{R_k} \ge s$
3. Keep at least the two biggest values per column: $r \leftarrow \text {max}(2,r)$
4. Keep all values of equal rank: while $C^i_{R_{r+1}} = C^i_{R_{r}}$ : $r \leftarrow r+1$
5. Round all values with ranks greater then *r* to zero, but keep those on the main diagonal and the first lower and first upper diagonals: $C^i_{R_k} \leftarrow 0$ for all *k* with $k > r \wedge R_k \notin \{(i-1, i, i+1) \, \text {mod} \, |S|\}$
6. Rescale the column to sum to 1: $C^i \leftarrow C^i/\text {sum}(C^i)$.

**Fig. 3:**
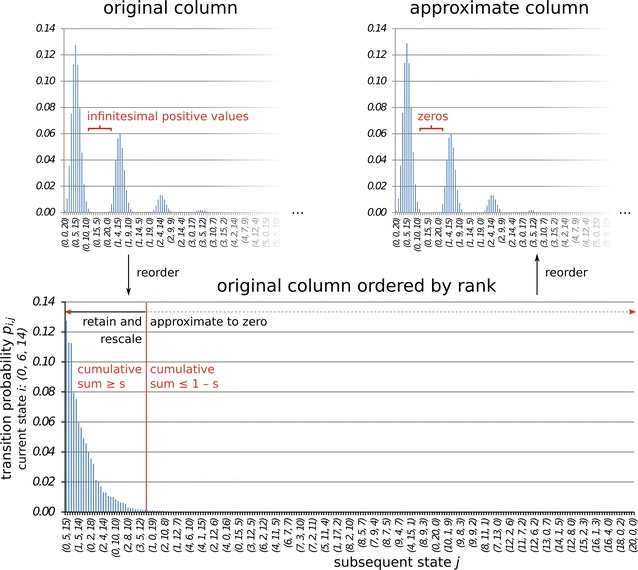
Illustration of the approximation algorithm $(s=0.99)$ for $N=20, \mu =10^{-6}, c=0.0$ and the state (0, 6, 14). Reordering is based on the relative size of the column entries and their index in the original column, respectively

The first two steps, together with the rounding in step five, form the core of the algorithm (compare Fig. 3), steps three and four prevent distortions and steps five and six ensure the continued validity of essential Markov chain transition matrix properties: Irreducibility is assured by keeping at least one outgoing and one incoming transition probability per state in such a way that all states remain connected (step five, first lower and first upper diagonal), aperiodicity by keeping all probabilities to stay at the same state (step five, main diagonal), and the rescaling of each column ensures left-stochasticity of the matrix (step six). In contrast, the property that one-step transitions are possible between all states is deliberately given up. The sparse approximation algorithm is available as the *appromatrix* function in the *mamoth* module.

Both the efficiency, i.e. the density or memory use of the resulting matrix, and the bias vary according to the value of *s* and the distribution of values in the original matrix. If *s* is low or the probability distribution in the column is far from uniform, more values will be discarded (compare Fig. 3). An appropriate value for *s* has to be determined heuristically by testing successively increasing values, up to the point where the bias due to the approximation no longer interferes with the interpretability of the model results. The sum of the differences between the entries of the approximate and original matrices has a theoretical upper limit of $(1-s) \cdot |S|$, but the effect of this perturbation on the model output may be more complex.

**Fig. 4:**
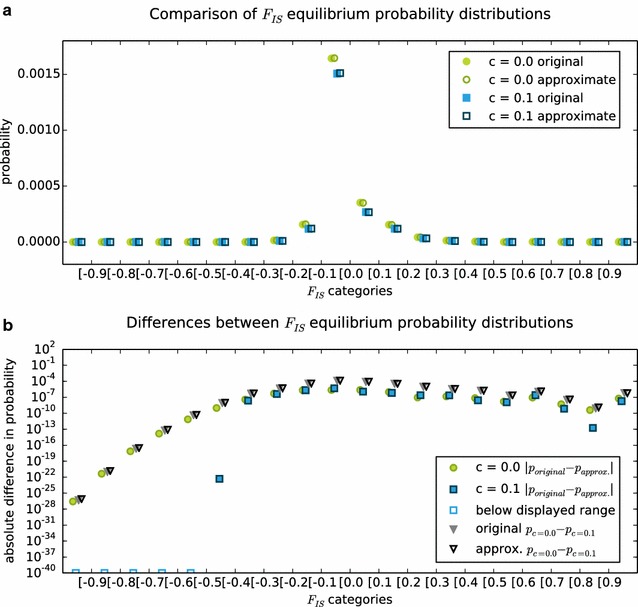
Comparison of the limiting distribution of $F_{IS}$ for $N=100, \mu =10^{-6}, c=\{0.0, 0.1\}$. **a** Probability distributions based on the original (*filled symbols*) and the approximate (*unfilled symbols*) matrix. **b** Pairwise differences between probability distributions, biologically interesting distances marked by *triangles*

In our model example, we analysed the effect of sparse approximation on the equilibrium $F_{IS}$ distribution derived from the dominant eigenvector of the transition matrix. The dominant eigenvector of either a sparse or dense matrix can be calculated with the *eigenone* function in *mamoth*, while a comparison between two vectors by a G-Test (correctly omitting infinity values from the test statistic) is implemented in the *testvector* function. A direct comparison between the “original” and “sparse approximate” equilibrium $F_{IS}$ distributions (Fig. 4) shows a very close fit which does not obscure the biologically relevant changes due to different rates of asexual reproduction. To test if the method gives similarly good results over a wider range of parameters (population size, mutation rate, rate of asexuality and approximation threshold), we performed a Global Sensitivity Analysis (GSA) [37, 38] using different divergence statistics to compare the limiting distribution of $F_{IS}$ derived from original and sparse approximate matrix [35, 39] and the density of the sparse matrix.

**Fig. 5:**
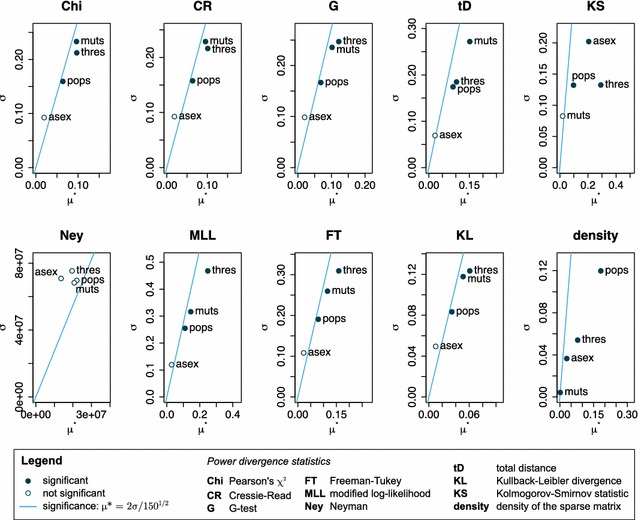
Global sensitivity analysis of original vs. approximate equilibrium $F_{IS}$ distribution. Absolute mean $\mu^{*}$ and standard deviation $\sigma$ of the elementary effects of population size *N* (pops), mutation rate $\mu$ (muts), rate of asexual reproduction *c* (asex) and sparse approximation threshold *s* (thres) on the density of the sparse approximate matrix, and on different statistics comparing the limiting $F_{IS}$ distributions derived from original and sparse approximate matrix. Based on 150 *Morris* samples from the parameter space: population size ($N = \{10, 20, \ldots , 100\}$), mutation rate ($\mu = \{10^{-12}, 10^{-11}, \ldots , 10^{-3}\}$), rate of asexual reproduction ($c = \{0.1, 0.2,\ldots , 1.0\}$) and approximation threshold ($s = \{0.8, 0.82, \ldots , 0.98\}$). Infinity values were omitted from the test statistic. The minimal upper bound of the parameters is one

The results of the GSA show that all four model parameters may generally have non-linear/interacting effects on the quality of the approximation, but in the mean these effects are not very strong (Fig. 5). Memory reduction is highly efficient as the mean density of the sparse matrices was only $\approx 0.11$. Individual densities ranged from $\approx 0.42$ (small matrix, high threshold) to $\approx 0.03$ (big matrix, low threshold), varying most strongly with the population size, though all four parameters have a significant influence. On our reference system (Intel Core i7-3930K 3.2 GHz processor with 64 GB RAM), calculating the sparse approximation based on the original matrix took on average 1.7 s for $N=50$ (14.6 s to construct the original), and 31.3 s for $N=100$ (221.7 s to construct the original). Finding the dominant eigenvector of sparse approximate and original matrix took on average 0.1 s (sparse) versus 51.7 s (original) for $N=50$ and 2.4 *s* (sparse) versus 7869.1 *s* (2 h, 11 min, 9.1 s, original) for $N=100$, so that in both cases less than one percent of the original runtime was needed with the sparse approximate matrix.

The overall similarity of the original and approximate equilibrium $F_{IS}$ distributions, measured with different divergence statistics (total distance, Kullback-Leibler divergence, power divergence statistics [40]; Fig. 5), is very high: e.g. the mean for the total distance $\sum \text {abs} (f_{orig}-f_{approx})$ is $\approx 0.06$. It is largely independent of the rate of asexual reproduction and depends most strongly on the approximation threshold and the mutation rate. In contrast, the maximal difference (Kolmogorov-Smirnov two-sample test statistic) between classes of the original and approximate equilibrium $F_{IS}$ distribution is hardly affected by the mutation rate, but rather by approximation threshold (high mean effect) and rate of asexual reproduction (strong non-linearity/interaction). Though on average not significant, the Kolmogorov-Smirnov test gave p-values below 0.05 in $20~\%$ of the parameter sets sampled. Consequently, the same approximation threshold can be used to compare the overall shape of the distributions across the whole range of rates of asexual reproduction, but it may have to be adapted if mutation rate and population size differ strongly between the modeled scenarios. Care must be taken when individual classes within the distribution (e.g. long-term fixation probability) shall be compared as the probabilities derived from a sparse approximate matrix may then be significantly different from the original.

In conclusion, sparse approximation using our algorithm has the advantage of being easily applicable to all transition matrices independently of the properties of the underlying model, and is well suited to provide an overview of the equilibrium $F_{IS}$ distribution under different rates of asexual reproduction in our model example. However, it needs an initial effort to verify the model results derived from the approximate matrix and to estimate their final bias. For fine-scale analyses, lumping states may provide an approximation-free alternative, but is not always possible as it depends on the model structure.

## Discussion

As the technological obstacles of working with “big data” become smaller, new opportunities arise especially for stochastic models, e.g. in population genetics. Yet these opportunities also lead to new challenges: results need to be brought into an interpretable form, and the technological boundaries further pushed back to allow even more complexity. We developed methods to help with the computational analysis and interpretation of state-rich time- and space-discrete Markov chain models in population genetics, focusing on the particularly challenging case of very dense matrices.

Markov chain models are a versatile framework also for population genetic questions, and may often provide a first step in the development of analytic formulae [3]. Further relevant parameters such as selection, migration or “unusual” reproductive systems can be easily included in such a model. Yet even for randomly mating population with genetic drift and mutation, a standard case of population genetics, a Markov chain model such as [16] may still yield additional information with the help of our visualization methods: In particular, the short-term dynamics, e.g. probabilistic trajectories connecting a current and a previous or predicted state, and the resulting variation around the expectation of convergence to the Hardy-Weinberg equilibrium are made visible. Especially for small populations, which are highly relevant e.g. for conservation genetics [41], and questions relating to development through time rather than just the long-term equilibrium, such Markov chain models may thus become valuable tools.

Though the size limitation for computational matrix analysis may never be completely removed, we showed that there are ways to circumvent it: even without access to specialized hardware, big, dense transition matrices may be manageable either by lumping states, or by approximating rare transitions to zero with our sparse approximation algorithm. In our model example, the approximation provided sufficiently accurate results for the limiting distribution of $F_{IS}$. Though there is an initial effort of verification, the advantage of sparse approximate matrices is considerable as they can subsequently be used also on less powerful hardware e.g. to speed up or allow the calculation of eigenvectors on systems incapable of storing the full model. In our model example, the size reduction of sometimes more than 90 % would e.g. make it possible to use the equilibrium $F_{IS}$ distributions for the inference of model parameters in an analysis software without having to store a—necessarily incomplete—reference collection of pre-calculated distributions for very big matrices. Moreover, some of our visualization methods (e.g. most probable neighbor, $p_{\text {in}}$, $p_{\text {stay}}$, $p_{\text {out}}$, *p*(*i*|*j*)) can be used without ever storing the whole matrix, while providing even very powerful conclusions about model behavior. Our sparse approximation method is not intended to substitute other approaches, and we did not test if it outperforms the accuracy of other approximations (e.g. diffusion approximation) for any specific question. Rather, it is a supplement, allowing to keep the structure of the original Markov chain model with the corresponding interpretation techniques beyond the technical limit, and a potential reference for the existing methods.

Individual-based models are becoming more and more popular in biology [42, 43], which will further increase the frequency of encountering computationally challenging cases such as the one we presented. In population genetics, modeling more complex evolutionary parameters such as life cycles and reproductive mechanisms, multi-dimensional fitness landscapes or dispersal may often lead to the necessity of extending the traditional models from allele frequencies [3] to genotypes. Due to the diploid/polyploid nature of most higher organisms, this will necessarily increase the size of transition matrices and equation systems to be analysed. By presenting our approach, we hope to encourage and inspire others to extend and adapt our methods, thus further paving the way for the use of Markov Chain models with big, dense transition matrices.

## Conclusion

We described and evaluated a set of tools, implemented in the Python module *mamoth*, for working with state-rich Markov chain models in population genetics. These tools ease the interpretation of model behavior by providing diagnostic visualizations of transition matrices, and allow substituting dense transition matrices with a sparse counterpart by applying an iterative approximation algorithm that is independent of model symmetry. Thus, our methods permit an advanced analysis of increasingly complex Markov chain models in population genetics, without giving up their space and time discrete structure. They may therefore contribute e.g. to the study of the population genetic consequences of partially clonal reproduction.

## Availability and requirements

The methods we described can be easily implemented in any scientific programming environment; we provide sample code for Python for all methods which do not rely on the specific state definitions of our model example.

*Project name:* mamoth.

*Project home page:*http://www6.rennes.inra.fr/igepp_eng/Productions/Software

*Operating system(s):* Platform independent.

*Programming language:* Python.

*Other requirements:* Python 2.7 or 3.4 and higher, extension modules numpy/scipy, matplotlib and networkx ([44– 46]).

*License:* GNU public license, version 2 (GPL2).

*Any restrictions to use by non-academics:* see GPL2 license.

## Additional files

10.1186/s13015-015-0061-5 Visualization algorithm runtimes. Runtimes in seconds for creating each subfigure of figures 1, 2 and Additional files 1–3. Means over three repetitions. Reference system: Intel Core i7-4850HQ 2.3 GHz processor, 16 Gb 1600 MHz DDR3 RAM.
10.1186/s13015-015-0061-5 Network display methods 1. Enlarged version of figure. Network display of transition matrices for $N=20, \mu =10^{-6}, c=0.0$. A. *De Finetti* diagram showing symmetry (dashed blue axis, red arrows corresponding to identical probabilities) and $F_{IS}$ isocurves (gray and black) B. $p_{stay}$ (node color), probability to stay at each state for one time step C. most probable path connecting (N,0,0) to (0,0,N) D. most probable neighbors (directed edges) and in-degree (node color), i.e. for each state the most likely outbound transition at the next time step and the number of inbound most likely transitions from other states.
10.1186/s13015-015-0061-5 Network display methods 2. Network display of transition matrices for $N=20, \mu =10^{-6}, c=0.0$. A. $p_{out}$ (node color), probability to leave this node in the next time step B. $p^{\infty }$ (node color), limiting probability of each state C. in-degree (node color) at flow between the fixation states (directed edges) D. $p_{in}$ (node color), probability to arrive each state if all previous states are equally probable.
10.1186/s13015-015-0061-5 Network display methods 3. Network display of transition matrices for $N=20, \mu =10^{-6}, c=0.0$. A. expected time to fixation (node color) according to start state B. *p*(*i*|(0, 15, 5)) (node color), probabilities of each state if the previous state was (0,15,5) C. $p^{\infty }_{in}$ (node color), probability to arrive at each state if the start state probabilities correspond to the limiting distribution D. betweenness-centrality (node color).
10.1186/s13015-015-0061-5 Landscape plot. Landscape plot of transition matrix for $N=20, \mu =10^{-6}, c=0.0$. Elevation rescaled by factor 5, color according to relative elevation (“valleys”: dark blue, “hills”: light grey). The lowest elevation equals zero, the reference *de Finetti* triangle is offset to -0.3.
