# Supplementary material for: Interpretation and approximation tools for big, dense Markov chain transition matrices in population genetics
Source: Algorithms Mol Biol. 2015 Dec 30;10:31. doi: 10.1186/s13015-015-0061-5 (PMC4696214; doi:10.1186/s13015-015-0061-5)
Supplement: Supplementary file 1 — 10.1186/s13015-015-0061-5 Visualization algorithm runtimes. Runtimes in seconds for creating each subfigure of figures 1, 2 and Additional files 1–3. Means over three repetitions. Reference system: Intel Core i7-4850HQ 2.3 GHz processor, 16 Gb 1600 MHz DDR3 RAM. [file 13015_2015_61_MOESM1_ESM.pdf]

Visualization method	subfigure	runtime [s]
Histogram	1-A	0.001
Histogram, logit(10)	1-B	0.001
Histogram, sparse approximation	1-C	0.055
Histogram, logit(10) reordered	1-D	0.211
De Finetti diagram	*A1-A / 2-A*	*0.405*
Most probable neighbor and in-degree	A1-D / 2-D	0.160
Probability to stay	A1-B / 2-B	0.231
Probability to arrive in an infinite run	A3-C	0.281
Probability to arrive from specified state	A3-B	0.288
Most probable path	A1-C / 2-C	0.339
Expected time to fixation	A3-A	0.380
Probability to arrive in one time step	A2-D	0.409
Limiting distribution (eigenvector)	A2-B	0.455
Probability to leave	A2-A	0.482
In-degree at percolation	A2-C	2.124
Betweenness-Centrality	A3-D	19.063
