# Supplementary material for: Interpretation and approximation tools for big, dense Markov chain transition matrices in population genetics
Source: Algorithms Mol Biol. 2015 Dec 30;10:31. doi: 10.1186/s13015-015-0061-5 (PMC4696214; doi:10.1186/s13015-015-0061-5)
Supplement: Supplementary file 2 — 10.1186/s13015-015-0061-5 Network display methods 1. Enlarged version of figure. Network display of transition matrices for $N=20, \mu =10^{-6}, c=0.0$N=20,μ=10-6,c=0.0. A. De Finetti diagram showing symmetry (dashed blue axis, red arrows corresponding to identical probabilities) and $F_{IS}$FIS isocurves (gray and black) B. $p_{stay}$pstay (node color), probability to stay at each state for one time step C. most probable path connecting (N,0,0) to (0,0,N) D. most probable neighbors (directed edges) and in-degree (node color), i.e. for each state the most likely outbound transition at the next time step and the number of inbound most likely transitions from other states. [file 13015_2015_61_MOESM2_ESM.pdf]

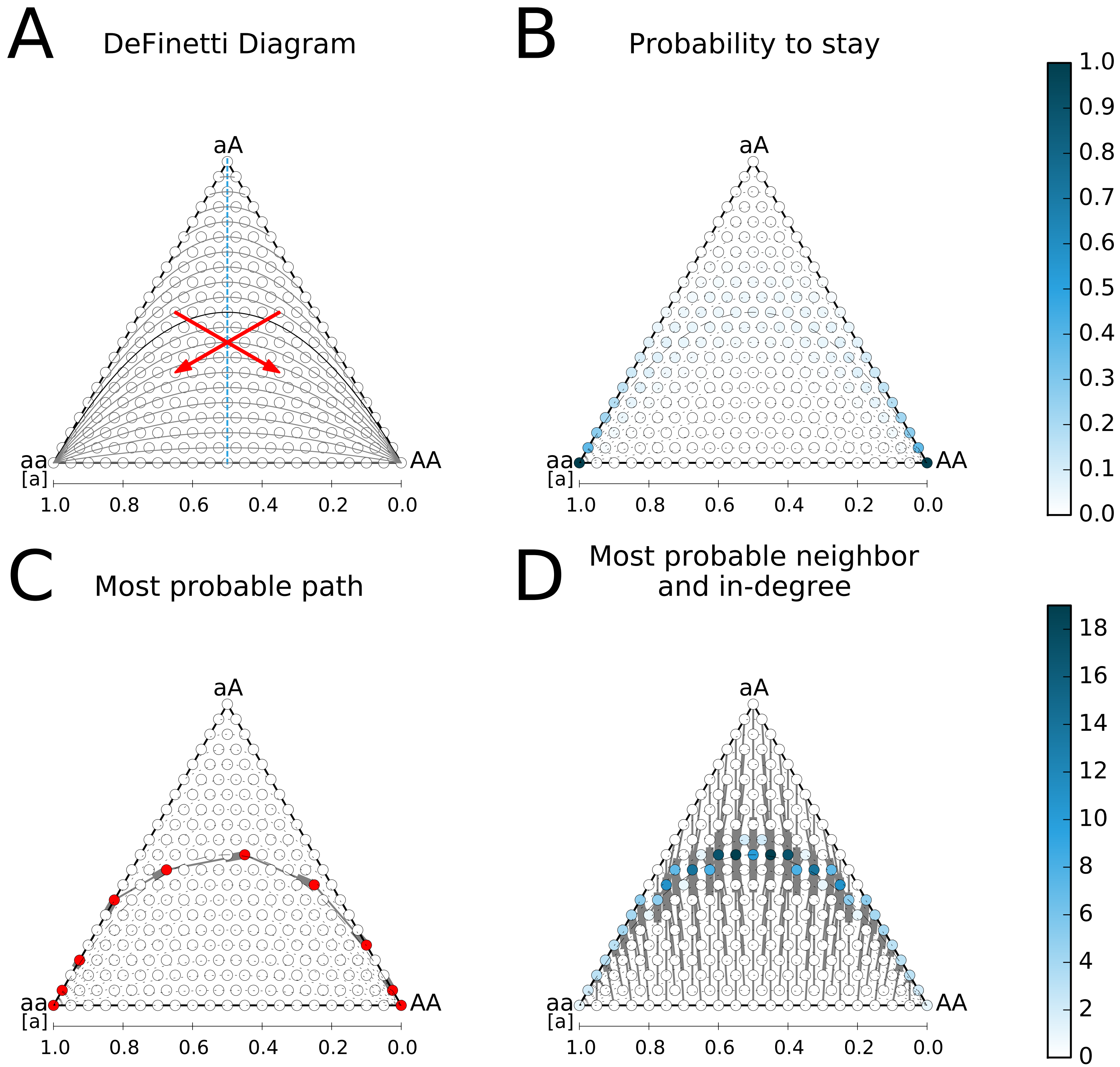
A
DeFinetti Diagram
B
Probability to stay
C
Most probable path
D
Most probable neighbor
and in-degree
aA
aa
AA
[a]
1.0
0.8
0.6
0.4
0.2
0.0
0.9
0.7
0.5
0.3
0.1
18
16
14
12
10
8
6
4
2
0
