# Supplementary material for: Interpretation and approximation tools for big, dense Markov chain transition matrices in population genetics
Source: Algorithms Mol Biol. 2015 Dec 30;10:31. doi: 10.1186/s13015-015-0061-5 (PMC4696214; doi:10.1186/s13015-015-0061-5)
Supplement: Supplementary file 3 — 10.1186/s13015-015-0061-5 Network display methods 2. Network display of transition matrices for $N=20, \mu =10^{-6}, c=0.0$N=20,μ=10-6,c=0.0. A. $p_{out}$pout (node color), probability to leave this node in the next time step B. $p^{\infty }$p∞ (node color), limiting probability of each state C. in-degree (node color) at flow between the fixation states (directed edges) D. $p_{in}$pin (node color), probability to arrive each state if all previous states are equally probable. [file 13015_2015_61_MOESM3_ESM.pdf]

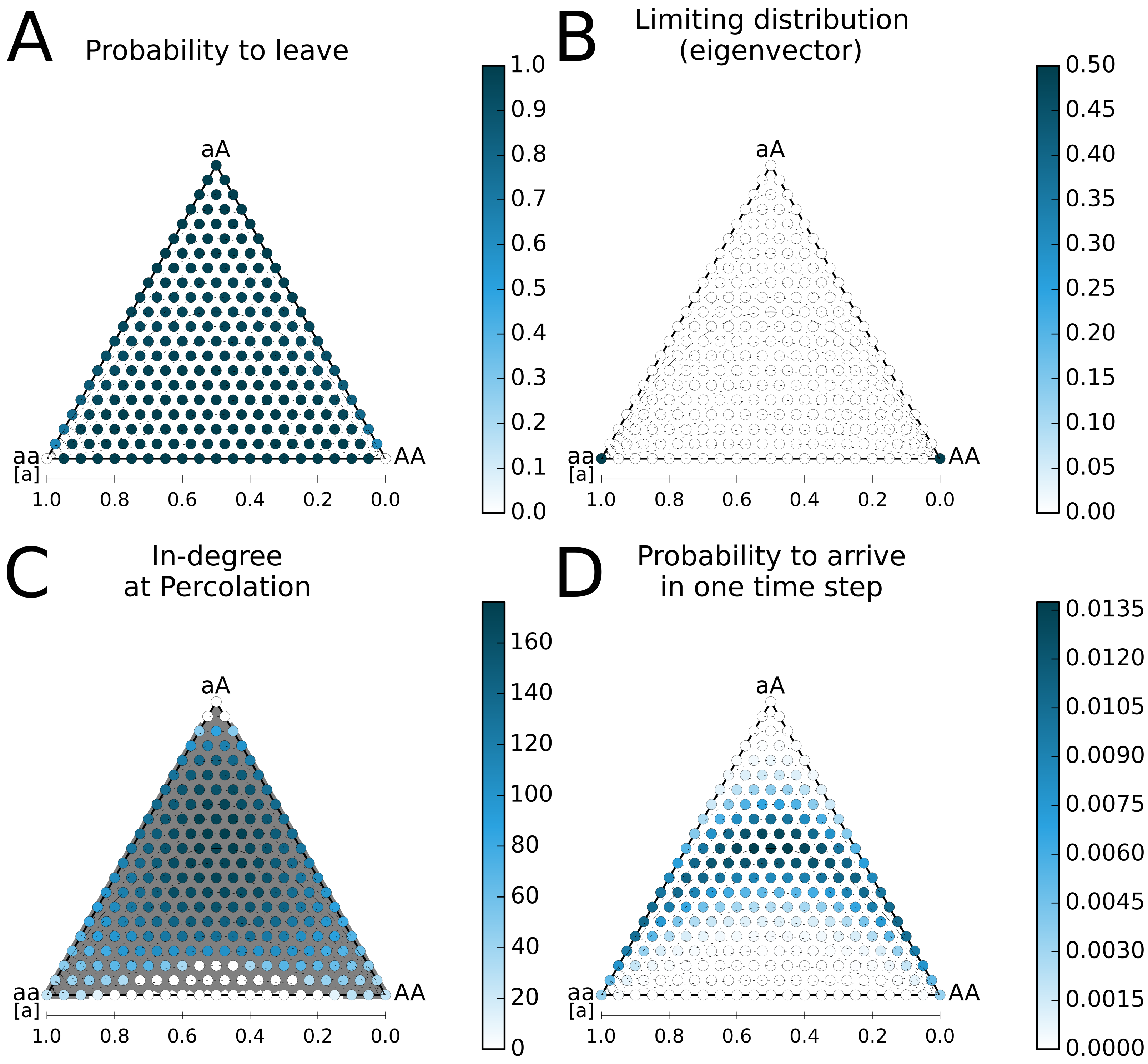
A
Probability to leave
1.0
0.9
0.8
0.7
0.6
0.5
0.4
0.3
0.2
0.1
0.0
aA
aa
AA
[a]
1.0
0.8
0.6
0.4
0.2
0.0
B
Limiting distribution
(eigenvector)
0.50
0.45
0.40
0.35
0.30
0.25
0.20
0.15
0.10
0.05
0.00
C
In-degree
at Percolation
160
140
120
100
80
60
40
20
0
D
Probability to arrive
in one time step
0.0135
0.0120
0.0105
0.0090
0.0075
0.0060
0.0045
0.0030
0.0015
0.0000
