# Supplementary material for: Interpretation and approximation tools for big, dense Markov chain transition matrices in population genetics
Source: Algorithms Mol Biol. 2015 Dec 30;10:31. doi: 10.1186/s13015-015-0061-5 (PMC4696214; doi:10.1186/s13015-015-0061-5)
Supplement: Supplementary file 4 — 10.1186/s13015-015-0061-5 Network display methods 3. Network display of transition matrices for $N=20, \mu =10^{-6}, c=0.0$N=20,μ=10-6,c=0.0. A. expected time to fixation (node color) according to start state B. p(i|(0, 15, 5)) (node color), probabilities of each state if the previous state was (0,15,5) C. $p^{\infty }_{in}$pin∞ (node color), probability to arrive at each state if the start state probabilities correspond to the limiting distribution D. betweenness-centrality (node color). [file 13015_2015_61_MOESM4_ESM.pdf]

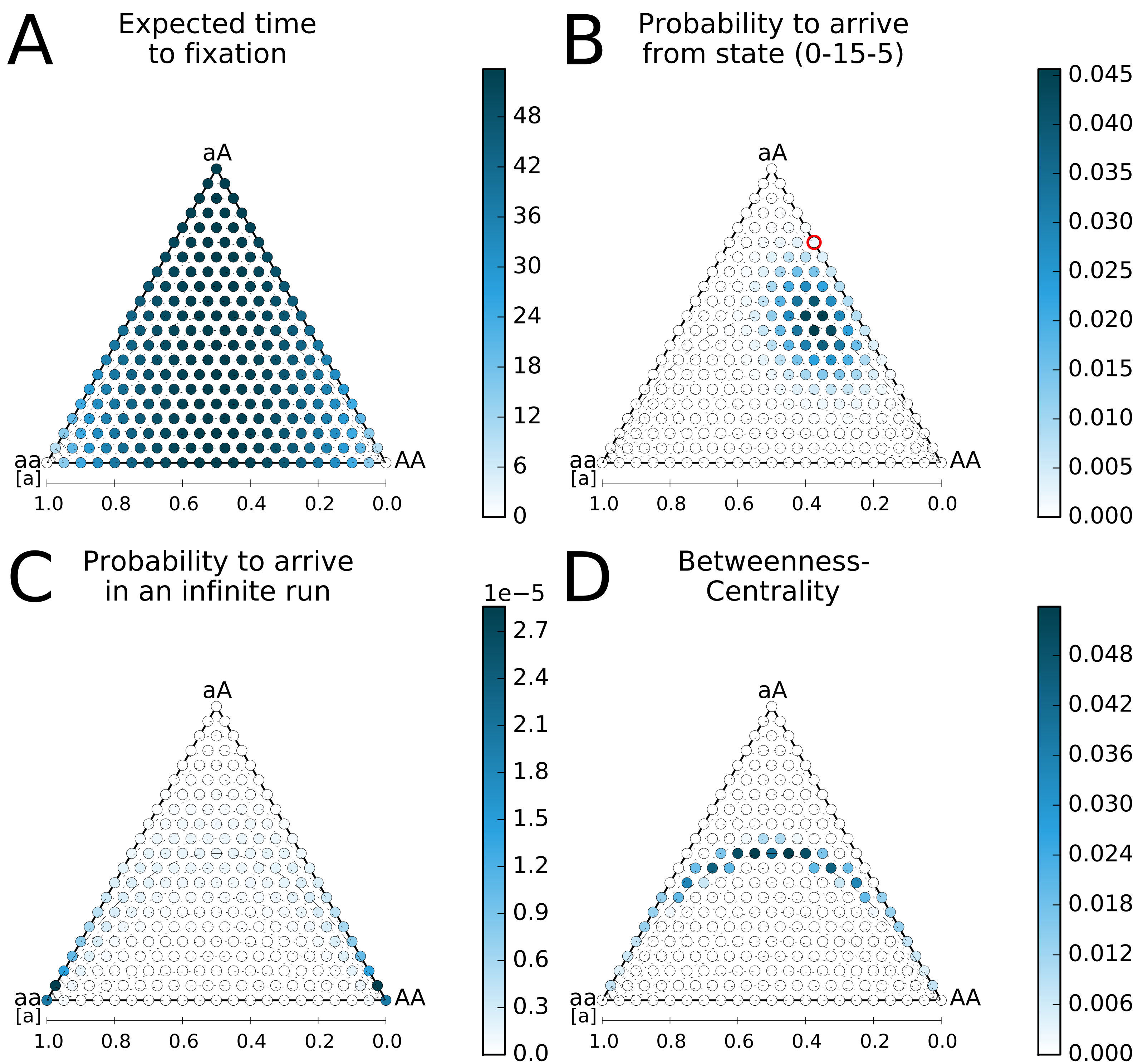
A
Expected time to fixation
B
Probability to arrive from state (0-15-5)
C
Probability to arrive in an infinite run
D
Betweenness-Centrality
aA
aa
AA
[a]
1.0
0.8
0.6
0.4
0.2
0.0
48
42
36
30
24
18
12
6
0
0.045
0.040
0.035
0.030
0.025
0.020
0.015
0.010
0.005
0.000
1e−5
2.7
2.4
2.1
1.8
1.5
1.2
0.9
0.6
0.3
0.0
0.048
0.042
0.036
0.030
0.024
0.018
0.012
0.006
0.000
