# Supplementary figures and images for: Interpretation and approximation tools for big, dense Markov chain transition matrices in population genetics
Source: Algorithms Mol Biol. 2015 Dec 30;10:31. doi: 10.1186/s13015-015-0061-5 (PMC4696214; doi:10.1186/s13015-015-0061-5)

# Landscape plot

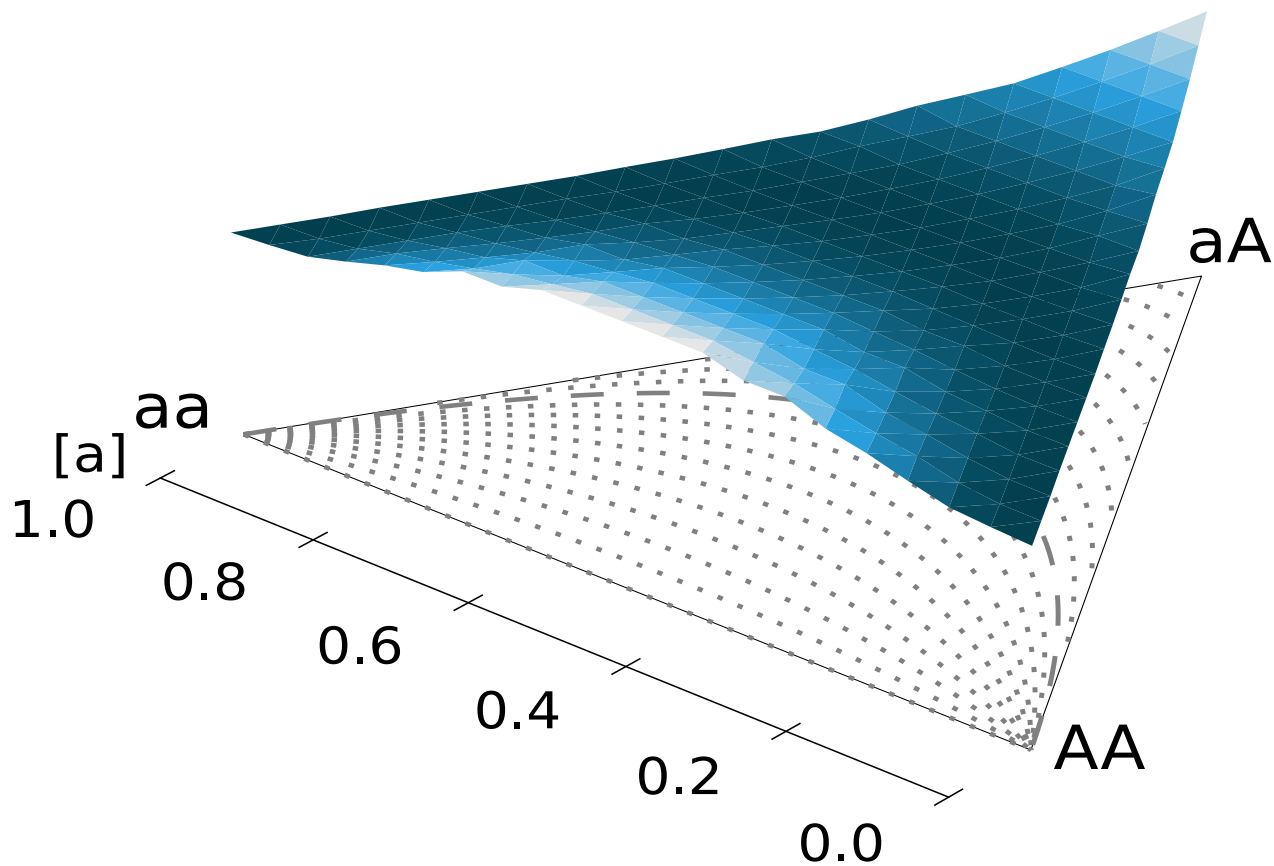

Supplement: Supplementary file 5 — 10.1186/s13015-015-0061-5 Landscape plot. Landscape plot of transition matrix for $N=20, \mu =10^{-6}, c=0.0$N=20,μ=10-6,c=0.0. Elevation rescaled by factor 5, color according to relative elevation (“valleys”: dark blue, “hills”: light grey). The lowest elevation equals zero, the reference de Finetti triangle is offset to -0.3. [file 13015_2015_61_MOESM5_ESM.pdf]
